# Technical Details of Laparoscopic Sleeve Gastrectomy Leading to Lowered Leak Rate: Discussion of 1070 Consecutive Cases

**DOI:** 10.1155/2017/4367059

**Published:** 2017-07-06

**Authors:** David L. Warner, Kent C. Sasse

**Affiliations:** University of Nevada, Reno School of Medicine, 75 Pringle Way, Suite 804, Reno, NV 89502, USA

## Abstract

**Introduction:**

Laparoscopic sleeve gastrectomy is a widely utilized and effective surgical procedure for dramatic weight loss in obese patients. Leak at the sleeve staple line is the most serious complication of this procedure, occurring in 1–3% of cases. Techniques to minimize the risk of sleeve gastrectomy leaks have been published although no universally agreed upon set of techniques exists. This report describes a single-surgeon experience with an approach to sleeve leak prevention resulting in a progressive decrease in leak rate over 5 years.

**Methods:**

1070 consecutive sleeve gastrectomy cases between 2012 and 2016 were reviewed retrospectively. Patient characteristics, sleeve leaks, and percent body weight loss at 6 months were reported for each year. Conceptual and technical changes aimed towards leak reduction are presented.

**Results:**

With the implementation of the described techniques of the sleeve gastrectomy, the rate of sleeve leaks fell from 4% in 2012 to 0% in 2015 and 2016 without a significant change in weight loss, as depicted by 6-month change in body weight and percent excess BMI lost.

**Conclusion:**

In this single-surgeon experience, sleeve gastrectomy leak rate has fallen to 0% since the implementation of specific technical modifications in the procedure.

## 1. Introduction

Sleeve gastrectomy has become the most widely performed bariatric surgical procedure, with an estimated 75,000 cases performed in 2013 in the United States [1]. Gastric leak remains the most serious complication and occurs in 1 to 3% of all cases and as high as 7% in one case series [2–5]. Gastric staple line leak occurs most commonly at the proximal aspect of the staple line and tends to be subacute in nature [5–9]. Leak is associated with a high degree of morbidity for the patient and cost of care for institutions and payers. Reported techniques to minimize occurrence of leak include changes in calibration tube size, changes in staple cartridge, use of fibrin glues, oversewing of staple line, and use of staple line reinforcement materials [3, 4, 10–12].

## 2. Methods

All cases of sleeve gastrectomy performed by a single surgeon were reviewed over a 5-year time period, under an IRB-approved protocol. A comprehensive review of the literature of sleeve gastrectomy leak was undertaken. We report 1070 consecutive cases of laparoscopic sleeve gastrectomy and the rate of gastric staple line leak over the time from January 1, 2012, to the end of 2016. The last cases included in the analysis took place in December of 2016 and were monitored for evidence of leaks through March of 2017.

All patients were evaluated with nutritional and psychological evaluations and medical and specialist evaluations in accordance with the nationally accredited center's protocol. Each patient underwent evaluation with either upper GI series or esophagogastroduodenoscopy and responded to clinical questions regarding the presence or absence of GERD symptoms. 18% of patients were diagnosed with hiatal hernia preoperatively and repaired concomitantly with the sleeve, and an additional 9% were diagnosed intraoperatively and repaired. In all cases, the baseline sleeve procedure was performed with laparoscopic technique. After insertion of four trocars, a Nathanson liver retractor was placed to elevate the left lateral segment of the liver. A bougie calibration tube was placed along the lesser curvature, and the greater curvature blood supply was divided with radiofrequency sealing, beginning 5 cm from the pylorus. Three Echelon green stapler cartridges were utilized in the antrum, using staple line reinforcement of bovine pericardium (Peristrips). The gastric body and fundus were stapled with varying Echelon stapler cartridges, which became consistent after 2014 with two gold cartridges in the mid body followed by two blue cartridges in the proximal fundus. The left crus was fully exposed. The most proximal stapler was angled 2-3 cm away from the esophagus. The hiatus was repaired with anterior cruroplasty without posterior dissection when a hiatal hernia less than 3 cm was present and with hiatal dissection and anterior and posterior cruroplasty when >3 cm. A methylene blue leak test was performed at the end of the procedure.

## 3. Results

Patient characteristics are detailed in Table 1. Over the course of 5 years, 1070 laparoscopic sleeve gastrectomy cases were performed, and a total of 14 leaks occurred (1.3%). During the time studied, the leak rate fell from a rate of 3.8% in 2012, to 3.7% in 2013, to 1% in 2014, and to 0% thereafter (Table 2). All of the leaks (100%) occurred within 3 cm of the gastroesophageal junction, on the proximal sleeve staple line. All of the leaks resolved after treatment with endoscopic stenting, or a combination of endoscopic treatments and surgical reoperation. There was no mortality among any of the 1070 cases, including all of the cases of leak, but one patient did experience a prolonged ICU stay and reoperative surgery with prolonged recovery of approximately 26 weeks. Weight loss results were compared for cases performed from 2012 to 2015, among the 84% of patients who had weight recorded at 6 months of followup after their sleeve procedure in 2012, 86% in 2013, 81% in 2014, and 88% in 2015. Weight loss results are reported as lost percentage of body weight (% BW) and percentage excess BMI lost (% EBMIL). Mean percent body weight loss at 6 months was 22%, 23%, 26%, and 24%, respectively, not significantly different from year to year (*p* = .34, ANOVA).

**Figure 1:**
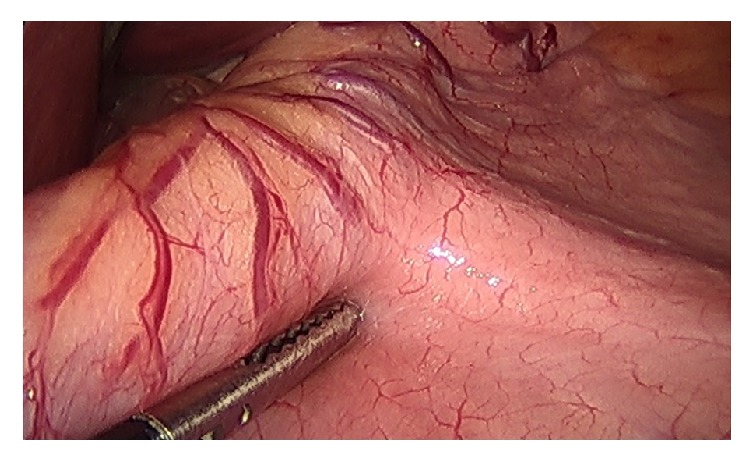
Stomach with 40-French sizing tube within the stomach, positioned along lesser curvature of stomach, in preparation for stapling.

**Figure 2:**
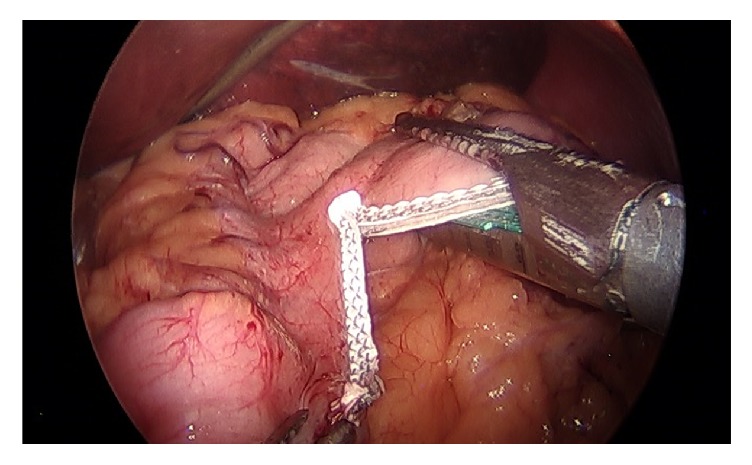
Maintaining a wide berth around bougie at incisura region.

**Figure 3:**
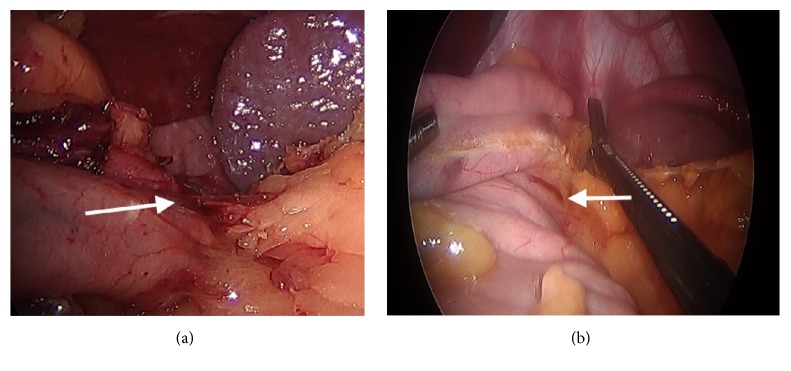
(a) Preserving the proximal posterior attachments and blood supply to the sleeve. (b) Preserving the proximal posterior attachments and blood supply to the sleeve.

**Figure 4:**
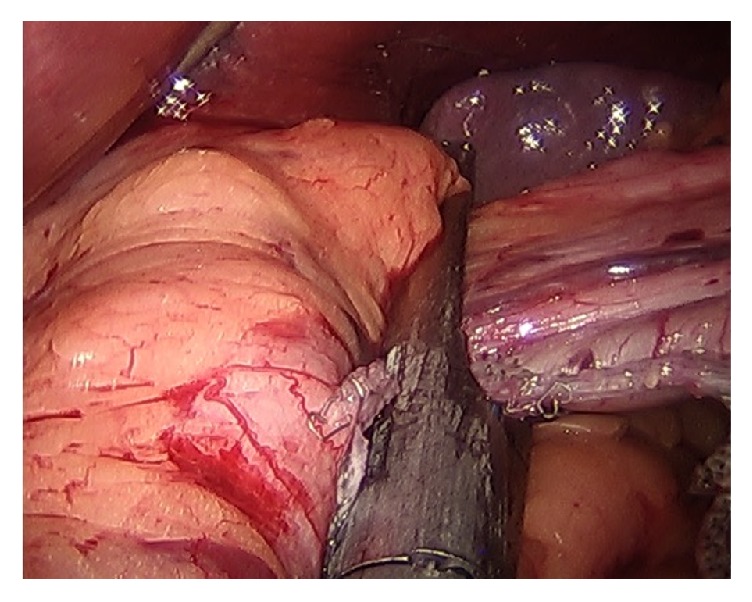
Final stapler loads using 3.5 mm staple height (Echelon Blue cartridge) without reinforcement material, angled to the left of the fat pad.

**Figure 5:**
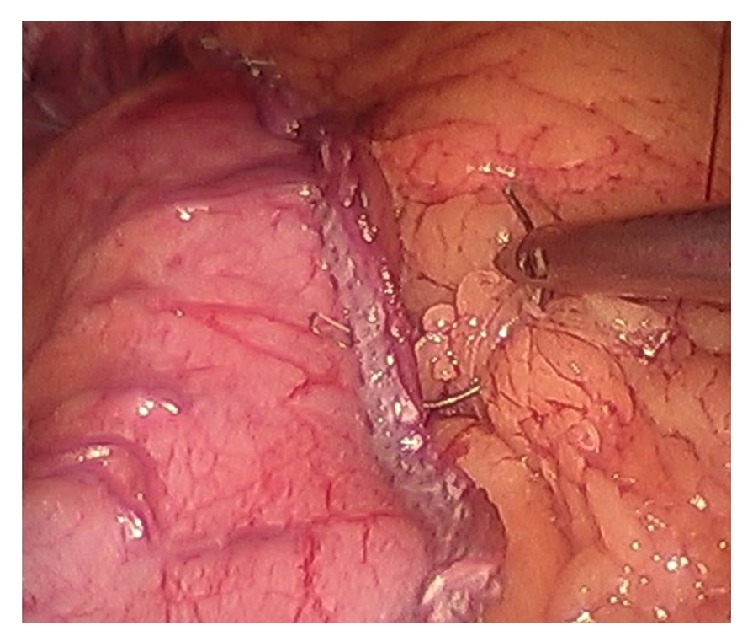
Suturing omentum back to sleeve staple line.

The identified technical elements during the change in leak rate from 3.8% to 0% were as follows:

1. Use of the 40-French sizing calibration tube (Figure 1).
2. Allowing generous volume around the sizing calibration tube at the curve of the incisura (Figure 2).
3. Avoidance of the disruption of cardiotuberosity branch arteries serving as the blood supply to the proximal stomach in the cardia region, especially posteriorly (Figure 3).
4. Angling the linear stapler to the left and more than 15 mm away from the true gastroesophageal junction.
5. Use of blue or 3.5 mm tissue stapler cartridges in the proximal stomach without staple line reinforcement (Figure 4).
6. Application of fibrin glue sealant (Tisseel, Baxter Corp.) to the staple line.
7. Hand-sewn, interrupted sutures to invert the staple line at the proximal 4 cm of the sleeve.
8. Apposition of omentum to rest in proximity to the completed staple line (Figure 5).
9. Suturing the omentum back to the mid and lower staple line to prevent a potentially obstructing “wind-sock” deformity.
10. Avoidance of 1-stage revisional sleeves concomitant with band removal.

**Figure 6:**
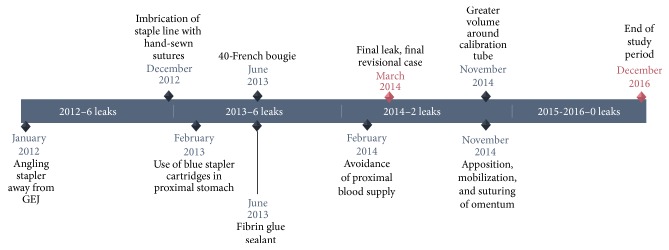
Timeline of technique implementation.

A timeline representing the occurrence of leaks and the implementation of the technical changes is displayed in Figure 6.

Each of the 13 revisional cases reported represents concomitant laparoscopic removal of a gastric band and conversion into a sleeve gastrectomy. One leak in each of the years 2013 and 2014 occurred in a revisional case, for a total leak risk of 2/13, or 15%, in revisional cases. The last leak occurred in March of 2014 in a revisional case, after which no further revisional band removals with sleeve were performed. Since March of 2014, over 650 consecutive laparoscopic sleeve procedures have been performed without a leak.

## 4. Discussion

Gastric leak following sleeve gastrectomy remains the most serious complication of sleeve gastrectomy. Leaks are most commonly subacute in nature and may present with an indolent course, weeks, or even months, after the procedure [5, 13]. Treatment includes establishing adequate drainage and utilizing endoscopic stents, endoluminal suturing, fibrin sealants, and surgical revision [14–21]. Numerous procedures may be required to resolve a sleeve leak, and morbidity and cost to the patient can be considerable.

The etiology of gastric sleeve leaks has been discussed and debated widely. In our center, all of the leaks from all sleeve cases in the past 8 years, whether performed at our center or outside centers and then transferred to our care, occurred in the proximal 4 cm or less of the sleeve. A high percentage of the published cases occur at this location [5–8]. Contributing factors include tissue ischemia, elevated intraluminal pressures, host impaired healing, and suboptimal closure techniques including poor stapler height choice, staple malformation, or hematoma formation. Blood supply has been long held as a key element in determining staple line and anastomotic integrity. The recent elegant cadaveric vascular anatomy study published by Perez demonstrates the fragility of the arterial blood supply to the proximal sleeve, the site of nearly all leaks. Specifically, the disruption of the posterior attachments of the proximal sleeve may be expected to disrupt cardiotuberosity branches of the left gastric artery [6]. It is evident from the anatomical study that the proximal sleeve is vulnerable to compromised blood supply stemming from the division of these small arterioles along the posterior wall of the proximal stomach. During surgery, it is often possible to see small vessels within the posterior attachments as the surgeon marches proximally along the sleeve (Figure 3). Preservation of those attachments and vessels may preserve important blood supply to the proximal sleeve and reduce the risks of leak.

Patient selection is often rightly cited as among the most important factors predictive of leaks and other complications. Tobacco use, steroid and medical immunosuppression, supermorbid obesity, NSAID use, diabetes, malnutrition, Crohn's disease, and revisional procedures have been associated with increased rates of gastric leak [21–23]. While we endeavor to modify risk factors which may be modified and screen out patients with prohibitive risks, we cannot as a practical matter turn away all patients with risk factors. Two of the 8 leak cases in the past 3 years involved a revisional procedure of removing a gastric band and converting into a sleeve. As a result of these cases, and others reported in the literature [22], we changed to a policy of staged conversions with a 6-month interval between band removal and sleeve gastrectomy and have experienced no leaks since.

Intraluminal pressure has been cited as a factor that may lead to increased leaks from the staple line, a logical contention and one that is supported by measurements of higher intraluminal gastric pressure within a sleeve than within a gastric pouch following roux-en-y gastric bypass [24, 25]. Gastric outlet obstruction and subsequent increased intraluminal pressure might be expected to promote staple line leak. Previously described stenosis, twist, or “wind-sock” deformity can each lead to gastric outlet obstruction, and each is prevented or minimized by the technique described [26]. Giving wide berth around the calibration tube or sizing bougie in the antrum and around the incisura minimizes narrowing, and suturing the omentum to the mid and distal staple line pexes the lower stomach to discourage twist or partially obstructing deformity.

Staple height and use of staple line reinforcement material have been widely debated in relation to their association with leak rate. While initial burst pressure is reduced when staple line reinforcement is used [27–29], there is conflicting evidence that reinforcement material reduces leak rates from staple lines in the more delayed time frame most common for gastric sleeve leaks [12]. Greater consensus is present for the finding that staple line bleeding is lessened with staple line reinforcement material, a problem most often encountered in the lower stomach and antrum. What is clear is that the tissue thickness at the proximal stomach is considerably thinner than in the antrum. Prior to 2012, our cases were performed with thick tissue loads (Echelon 4.1 mm, Ethicon Corp.) with staple line reinforcement of the distal stomach using bovine pericardium (Peristrips). Staple height diameter of 3.5 mm for the proximal sleeve has been considered the most appropriate by the sleeve gastrectomy working group [5] and use of this thinner staple height (Echelon 3.5 mm, Ethicon Corp.) without staple line reinforcement at the proximal sleeve has been an element of the technique of 0% leaks.

Calibration tube size remains a debated topic among bariatric surgeons, with the consensus panel recommending a bougie size between 32 Fr and 40 Fr [5]. Some authors have reported greater weight loss success with smaller bougie size, and some authors have noted the association of increased complications of both leak and stenosis with smaller bougie size [3]. Because staple line leaks carry such a high cost in terms of morbidity and health care expense, we have taken the approach that preventing such leaks is of paramount importance. In 2013, the sleeve gastrectomy procedures were performed with 34 Fr bougies; in 2014 a mix of 34 Fr and 40 Fr bougie sizes was utilized, and in 2015 and 2016 all cases were performed with a 40 Fr bougie with 0 leaks.

Fibrin sealants have been promoted for their effectiveness at reducing bleeding from a variety of surgical tissues. Cottam et al. reported the successful use of Tisseel in achieving a 1.6% leak rate in a series of 126 high-BMI individuals undergoing sleeve gastrectomy [30]. There is likely little adverse effect from application of fibrin sealants, and indirect evidence from studies of hepatic and pancreatic tissues, that leaks could potentially be reduced by application of fibrin sealants (Tisseel, Baxter Corp.) [31, 32].

Omental apposition, or omentoplasty, to the staple line may be thought of as a technique which takes a page from historical surgical repairs of perforations of the stomach using an omental patch, a widely successful technique [33]. In some cases, the attachments of the omentum to the spleen or to the abdominal sidewall prevent the omentum from freely migrating to the proximal staple line, and in such cases we believe it makes sense to free the omentum so that it may do so, while maintaining its blood supply.

The role of concomitant hiatal hernia repair, choice of repair technique, and whether or not to reinforce crural repair continue to be defined in the bariatric literature. Increased dissection at the hiatus and angle of His might theoretically exacerbate the already diminished perfusion of the proximal gastric fundus which has been observed among obese individuals [34]. In published studies, there has been no clear relationship between leak rate and performance of concomitant hiatal hernia repair [35, 36] and a recent prospective study in which 96 patients underwent complete GE junction mobilization, exposure, hiatoplasty, and concomitant sleeve resulted in 0 leaks [37]. In this series, repair of hiatal hernia did not appear to influence risk of leak. The optimal role of hiatal hernia repair in sleeve gastrectomy remains to be defined.

“Learning curve” may describe intangible improvements that come from experience and result in reduced complications over time. As has been done with other studies [38], this paper is an attempt to identify granular elements of “learning curve” that may have contributed to reduction in sleeve leaks over time.

Each element may be debated for its contribution to the reduced leak rate, and future studies will undoubtedly shed further light as to which of these elements is simply unnecessary to still achieve a 0% leak rate. It can be argued that, in this series, little undefined learning curve mystery played a role since the surgeon began performing sleeve gastrectomy in 2009 after having performed over 2000 laparoscopic roux-en-y gastric bypass cases. Little change has occurred with assistants and staff over the past 7 years. It remains to be seen if some of these technical elements—namely, the change to a 40 Fr bougie and the reduced tightness around the bougie in the distal stomach and incisura region—will result in compromised weight loss over a longer time period. If this proves to be the case, then we face a complex discussion over the trade-offs of reduced complications versus maximal weight loss results in sleeve gastrectomy. We consider it likely that innovative revisional and medically guided weight loss options including endoscopic revisions, dietary programs, and prescription medication therapy may mitigate differences in weight loss which could potentially occur over a longer time horizon as a result of the technical changes described herein to prevent leak.

Fear of complications remains the most widely cited objection to bariatric surgery referrals among referring doctors, and it remains the most widely cited objection to bariatric surgery among patients who most need it [39]. If we are to bring this life-saving intervention to more individuals, then we must relentlessly reduce complications, the most serious of which is leak in sleeve gastrectomy. The primary weakness of this paper is that these technical elements are offered in an observational manner and each is not rigorously compared in a randomized fashion. It is hoped that identifying these elements in such a granular fashion may help spur more detailed examinations of the optimal methods to reduce complications.

## 5. Conclusion

In this series over a 5-year period, a sequence of technical changes was made in an effort to prevent the most serious complication of sleeve gastrectomy, namely, staple line leak. Collectively, these technical elements have succeeded in reducing the leak rate from 3.8% in 2012 to 0% in 2015 and 2016 without a change in the 6-month weight loss results. More than 600 consecutive sleeve gastrectomy procedures have now been performed without a leak.

## Figures and Tables

**Table 1:** Sleeve gastrectomy patient characteristics from 2012 to 2016.

	2012	2013	2014	2015	2016
*N*	158	164	188	240	320
Female (%)	117 (74)	126 (77)	137 (73)	175 (75)	227 (71)
Male (%)	41 (26)	38 (23)	51 (27)	58 (25)	93 (29)
Mean age (years)	37.7	38.2	39.6	38.9	37.4
Mean weight (kg)	125	126	131	128	133
BMI (kg/m^2^)	46	47	49	48	47
Revisional	4	4	5	0	0

**Table 2:** Leak incidence and weight loss results.

Year	Sleeve cases	Leaks	Percent leaks	Initial BMI (kg/m^2^)	BMI at 6 mo. (kg/m^2^)	Change in BMI (kg/m^2^)	6 mo. Wt. loss change in percentage of body weight (% BW)	6 mo. percent excess BMI lost (% EBMIL)
2012	158	6	3.80%	46	36	10	22%	48%
2013	164	6	3.70%	47	36	11	23%	49%
2014	188	2	1.00%	49	36	13	26%	53%
2015	240	0	0%	48	37	11	24%	50%
2016	320	0	0%	47	—	—	—	—
